# Oxidative Stress, Energy Metabolism Disorder, Mitochondrial Damage, and miR-144 Participated in Molecular Mechanisms of 4-Octylphenol-Caused Cardiac Autophagic Damage in Common Carps (*Cyprinus carpio* L.)

**DOI:** 10.3390/metabo15060391

**Published:** 2025-06-11

**Authors:** Minna Qiu, Chunyu Jiang, Jiatian Liang, Qin Zhou, Yuhao Liu, Zhiyu Hao, Yuhang Liu, Xiumei Liu, Xiaohua Teng, Wei Sun, You Tang

**Affiliations:** 1 College of Animal Science and Technology, Northeast Agricultural University, Harbin 150030, China; 18800427032@163.com (M.Q.); 18103648930@163.com (C.J.); 13945431738@163.com (J.L.); 18745100883@163.com (Q.Z.); 15146551036@163.com (Y.L.); haozhiyu9@gmail.com (Z.H.); a73140050020220221@163.com (Y.L.); tengxiaohua@neau.edu.cn (X.T.); 2 College of Life Sciences, Yantai University, Yantai 264005, China; xiumei0210@163.com; 3 College of Information Technology, Jilin Agricultural University, Changchun 132101, China; 4 College of Electrical and Information Engineering, Jilin Agricultural Science and Technology University, Jilin 132101, China; 5 Research and Development Department, Jilin Dianchuang Information Technology Co., Ltd., Changchun 130117, China

**Keywords:** 4-OP, oxidative stress, energy metabolism disorder, mitochondrial dynamics imbalance, autophagy

## Abstract

**Background/Objectives**: In 4-octylphenol (4-OP), a toxic environmental pollutant with endocrine disruptive effect, the use of 4-OP causes pollution in the freshwater environment and poses risks to aquatic organisms. Common carps (*Cyprinus carpio* L.) live in freshwater and are experimental animals for studying the toxic effects of environmental pollutants on fish. Its heart is susceptible to toxicants. However, whether 4-OP has a toxic effect on common carp heart remains unknown. **Methods**: Here, we conducted a common carp 4-OP exposure experiment (carp treated with 17 μg/L 4-OP for 45 days), aiming to investigate whether 4-OP has a toxic effect on common carp hearts. We observed the microstructure and ultrastructure of carp heart and detected autophagy genes, mitochondrial fission genes, mitochondrial fusion genes, glycolytic enzymes, AMPK, ATPase, and oxidative stress factors, to investigate the molecular mechanism of 4-OP induced damage in common carp hearts. **Results**: Our results showed that 4-OP exposure caused mitochondrial damage, autophagy, and damage in common carp hearts. 4-OP exposure increased the levels of miR-144, and eight autophagy factors (Beclin1, RB1CC1, ULK1, LC3-I, LC3-II, ATG5, ATG12, and ATG13), and decreased the levels of four autophagy factors (PI3K, AKT, mTOR, and SQSTM1). Furthermore, 4-OP exposure induced the imbalance between mitochondrial fission and fusion and mitochondrial dynamics imbalance, as demonstrated by the increase in three mitochondrial fission factors (Mff, Drp1, and Fis1) and the decrease in three mitochondrial fusion factors (Mfn1, Mfn2, and Opa1). Moreover, excess 4-OP treatment caused energy metabolism disorder, as demonstrated by the reduction in four ATPase (Na^+^K^+^-ATPase, Ca^2+^Mg^2+^-ATPase, Ca^2+^-ATPase, and Mg^2+^-ATPase), elevation in four glycolysis genes (HK1, HK2, LDHA, and PGK1), reduction in glycolysis gen (PGAM2), and the elevation in energy-sensing AMPK. Finally, 4-OP treatment induced the imbalance between antioxidant and oxidant and oxidative stress, as demonstrated by the increase in oxidant H_2_O_2_, and the decreases in five antioxidant factors (CAT, SOD, T-AOC, Nrf2, and HO-1). **Conclusions**: miR-144 mediated autophagy by targeting PI3K, mTOR, and SQSTM1, and the miR-144/PI3K-AKT-mTOR/ULK1 pathway was involved in 4-OP-induced autophagy. Mff-Drp1 axis took part in 4-OP-caused mitochondrial dynamics imbalance, and mitochondrial dynamics imbalance mediated autophagy via Mfn2-SQSTM1, Mfn2/Beclin1, and Mff-LC3-II axes. Energy metabolism disorder mediated mitochondrial dynamics imbalance through the AMPK-Mff-Drp1 pathway. Oxidative stress mediated energy metabolism disorder via the H_2_O_2_-AMPK axis. Taken together, oxidative stress triggered energy metabolism disorder, induced mitochondrial dynamics imbalance, and caused autophagy via the H_2_O_2_-AMPK-Mff-LC3-II pathway. Our study provided references for the toxic effects of endocrine disruptor on common carp hearts, and provided a basis for assessing environmental pollutant-induced damage in common carp heart. We only studied the toxic effects of 4-OP on common carp, and the toxic effects of 4-OP on other fish species need to be further studied.

## 1. Introduction

4-Octylphenol (4-OP), a toxic endocrine disruptor with estrogenic activity, is used in the production of chemical industrial products, such as surfactants, adhesives, emulsifiers, rubber-processing chemicals, and fuel stabilizers, which resulted in 4-OP entering the freshwater environment. 4-OP was found in the raw wastewater (untreated influent wastewater) of five wastewater treatment plants in Saskatchewan, Canada; the concentration of 4-OP was 288.7–322.9 ng/L in one plant and 24.2–141.6 ng/L in another [1]. Surface water was collected from 14 sampling sites in the Three Gorges Reservoir Region of China, and 4-OP was detected in 10 sampling points, with concentrations ranging from 11 to 120 ng/L [2]. In Brazil, 4-OP was detected at concentrations of 80–124 ng/L in tap water and at concentrations of 5–17 μg/L in the Iraí River [3]. It is worrying that 4-OP in the environment entered and accumulated in aquatic organisms and humans, which may pose a potential threat to aquatic organisms and human health. In Taizhou, China, a 4-OP concentration of 9.50–10.87 ng/g was found in Coastal Mud Shrimp [4]. Among 96 fish samples from Terengganu River in Malaysia, 4-OP was found in 22 *Arius* sp. samples with concentrations of 10.52–26.48 ng/g [5]. In 59 human milk samples in Taiwan, China, 4-OP was found in 18 samples, and the 4-OP detection rate and 4-OP concentrations were 30.5% and 1.16–2.97 μg/kg, respectively [6]. Thus, we wanted to investigate the toxic mechanisms of freshwater fish in the 4-OP exposure condition.

Three studies reported that 4-OP had toxic effects on freshwater fish. 4-OP exposure reduced the survival rate of juvenile goldfish (*Carassius auratus*) [7], caused damage in primary hepatocytes derived from pearl mullet (*Alburnus tarichi*) [8], and in common carp gill [9]. However, whether 4-OP has negative effects on fish hearts remains unknown. In humans, exposure to environmental endocrine disruptors has been linked to an increased risk of cardiovascular disease [10]. Two research studies showed that the isomers of 4-OP had toxic effects on fish hearts. Exposure to the linear isomer of 4-OP, 4-nonylphenol (4-NP), resulted in a decreased heart rate in medaka (*Oryzias latipes*) [11]. The branched isomer of 4-OP, 4-tert-octylphenol (4-t-OP), was shown to reduce heart rate and induce cardiac damage and cardiovascular abnormalities in zebrafish (*Danio rerio*) [12]. Hence, we hypothesized that 4-OP exposure can cause cardiac damage in fish. Common carp is an omnivorous fish and a commonly eaten freshwater fish in daily life. Common carp is also a commonly used experimental animal in fish toxicology research. Therefore, we wanted to use common carp as a research object to investigate whether 4-OP exposure can cause cardiac injury in common carp.

The heart requires a continuous energy supply, and ATP serves as the primary energy source. ATP is hydrolyzed by ATPase to release energy, maintaining normal cardiac function. Research showed that toxic endocrine disruptors altered ATPase and disrupted energy metabolism in fish. Tao et al. (2020) demonstrated that monobutyl phthalate changed ATPase (Na^+^-K^+^-ATPase, Ca^2+^-ATPase, and Mg^2+^-ATPase) and induced energy metabolism disturbance in zebrafish gills [13]. In common carp, cadmium (Cd) exposure resulted in altered activities of Na^+^-K^+^-ATPase, Ca^2+^-ATPase, Ca^2+^Mg^2+^-ATPase, and Mg^2+^-ATPase, and damaged heart [14]. However, whether 4-OP can cause ATPase activity changes in common carp remains unknown. Notably, AMPK, an energy sensor, plays an important role in regulating the heart’s energy metabolism. Mukherjee et al. (2022) reported that toxic endocrine disruptor nonylphenol exposure caused injury and altered AMPK in zebrafish livers [15]. However, it is unknown whether 4-OP can affect AMPK in common carp. Glycolysis is one of the basic pathways of energy metabolism and can provide energy to the heart. Research studies found that toxic endocrine disruptors caused poisoning in animals, and glycolysis factors were altered. In Cd-treated largemouth bass (*Micropterus salmoides*), the testis was injured, and lactate dehydrogenase A (LDHA) changed [16]. Bisphenol AF exposure caused changes in glycolysis enzymes hexokinase 1 (HK1) and hexokinase 2 (HK2), and inhibited glycolysis in zebrafish (*Danio rerio*) muscle [17]. Lipid deposition and phosphoglycerate kinase 1 (PGK1) change were observed in BPA-treated Rare Minnow *Gobiocypris rarus* livers [18]. In Cd-treated mice, phosphoglycerate mutase 2 (PGAM2) was altered and glycolysis was interrupted [19]. However, whether 4-OP can affect glycolysis in common carp remains unknown. Thereby, we hypothesized that 4-OP can induce energy metabolism disorder in terms of ATPase activity, AMPK, and glycolysis in common carp hearts.

Mitochondria are the most abundant organelle in cardiomyocytes, and most of the energy required by the heart is produced by mitochondria. The balance of mitochondrial dynamics is crucial for maintaining normal mitochondria and cardiac function. Research reported that toxic endocrine disruptors altered mitochondrial fission and mitochondrial fusion indicators and caused mitochondria damage in fish. In mono (2-ethylhexyl) phthalate (MEHP)-treated ctenopharyngodon idellus kidney (CIK) cells, mitochondrial fission factors (multitranche financing facility (Mff), dynamin-related protein 1 (Drp1)), mitochondrial fusion factors (mitofusin-1 (Mfn1), mitofusin-2 (Mfn2)), and optic atrophy 1 (OPA1) changed, and mitochondria were damaged [20]. After zebrafish (*Danio rerio*) were exposed to dibutyl phthalate, mitochondrial fission factor (Fis1) and fusion factors (Mfn1, Mfn2, and OPA1) were altered, as mitochondria damage was induced [21]. Liu et al. (2023) detected mitochondria fission factors (Drp1 and Mff) and mitochondrial fusion factors (Mfn1, Mfn2, and OPA1) in common carp hearts, and found mitochondria damage after Cd treatment [14]. However, whether 4-OP exposure can alter mitochondrial fission factors and mitochondrial fusion factors, and damage mitochondria in common carp heart, remains unclear. Therefore, we wanted to measure mitochondrial fission factors (Mff, Drp1, and Fis) and mitochondrial fusion factors (Mfn1, Mfn2, and OPA1).

Interestingly, oxidative stress occurred and energy homeostasis was dysregulated in zebrafish liver following nonylphenol exposure [15]. After endocrine disruptor 4-tert-butylphenol (4-tBP) exposure, oxidative stress occurred and mitochondria were damaged in common carp livers [22]. Thereby, we also wanted to explore if 4-OP exposure can induce oxidative stress in common carp. Several studies discovered that endocrine disruptors affected the oxidative indicator (hydrogen peroxide (H_2_O_2_)) and anti-oxidative indicators (catalase (CAT), superoxide dismutase (SOD), and total antioxidant capacity (T-AOC), nuclear factor erythroid 2-related factor 2 (Nrf2), and heme oxygenase-1(HO-1)), and induced oxidative stress in fish. 4-tBP treatment caused H_2_O_2_, CAT, SOD, and T-AOC changes and induced oxidative stress in common carp head kidneys [23]. Desai et al. (2023) demonstrated that Nrf2 altered and oxidative stress occurred in the brain of zebrafish treated with 4-nonylphenol [24]. In 1,2,3,7,8-polychlorinated dibenzo-p-dioxin-treated primary Atlantic cod hepatic cells, HO-1 changed and oxidative stress occurred [25]. It was reported that 4-OP exposure changed SOD and induced oxidative stress in primary cultured hepatocytes derived from pearl mullet (*Alburnus tarichi*) [8]. However, whether 4-OP exposure can induce oxidative stress in common carp is still unclear. Thus, we wanted to investigate whether 4-OP exposure can induce oxidative stress in common carp hearts by measuring oxidation (H_2_O_2_) and anti-oxidation (CAT, SOD, T-AOC, Nrf2, and HO-1) factors.

In common carp livers exposed to endocrine disruptor 2,4-di-tert-butylphenol, Xie et al. (2024) measured oxidative stress factors (CAT and SOD) and autophagy factors (Beclin1, microtubule-associated protein 1A/1B-light chain 3-I (LC3-I), microtubule-associated protein 1A/1B-light chain 3-II (LC3-II), autophagy-related 5 (ATG5), mechanistic target of rapamycin (mTOR), and sequestosome 1 (SQSTM1)), and found that oxidative stress and autophagy occurred [26]. Several studies found that autophagy was involved in the toxic mechanism of endocrine disruptors in human cell lines and fish. Mahemuti et al. (2018) showed that autophagy factors (RB1 inducible coiled-coil 1 (RB1CC1) and sequestosome 1 (SQSTM1)) altered, autophagy occurred, and cells were damaged in BPA-treated human fetal lung fibroblasts [27]. In MEHP-treated CIK cells, autophagy factors (Beclin1, autophagy activating kinase 1 (ULK1), LC3-II, autophagy-related 12 (Atg12), and autophagy-related 13 (Atg13)) altered, autophagy occurred, and cells were damaged [20]. In synthetic phenolic antioxidants-treated grass carp, autophagy factors (Beclin1, LC3-I, protein kinase B (AKT), phosphatidylinositol 3-kinase (PI3K), and mTOR) changed, and autophagy and damage occurred in livers [28]. After Cd treatment, autophagy factors (Beclin1, ULK1, LC3-I, LC3-II, autophagy-related 5 (ATG5), ATG12, mTOR, and SQSTM1) changed, inducing autophagy and damage in common carp hearts [14]. However, no studies have reported whether excessive 4-OP can trigger cardiac autophagy in fish. Therefore, we assumed that 4-OP can affect autophagy indicators and induce autophagy in fish. MicroRNAs (miRNAs), a class of endogenous, small, and non-coding single-stranded RNAs [29], can negatively regulate gene expression by targeting mRNA. miRNAs can be involved in the molecular mechanism of autophagy induced by endocrine disruptors via targeting autophagy genes in common carp [14,30]. A miRNA can participate in the physiological functions of an organism by targeting multiple genes. It is noted that our previous transcriptome sequencing analysis indicated that miR-144 was differentially upregulated in 4-OP-teated common carp (unpublished data, as shown in Table S1). We further predicted that three autophagy genes (PI3K, mTOR, and SQSTM1) were target genes of miR-144 using a bioinformatics website (http://www.bioinformatics.com.cn/, accessed on 10 January 2025). Accordingly, we wanted to study whether miR-144 was involved in the molecular mechanism of 4-OP-caused autophagy in common carp hearts via targeting PI3K, MTOR, and SQSTM1.

In summary, we wanted to investigate the effect of 4-OP exposure on common carp, to explore the complex mechanisms of 4-OP-induced cardiotoxicity from the perspectives of ATPases, AMPK, glycolysis enzymes, mitochondrial dynamics, OS, autophagy, and miR-144. Our study will offer novel foundation knowledge on assessing the risks of 4-OP exposure and investigating endocrine disruptor-induced toxicity in common carp.

## 2. Materials and Methods

### 2.1. Animal Ethics and Animal Sources

All procedures in this study were approved by the Animal Care and Use Committee of Northeast Agricultural University (No. NEAUEC20240229). Eighty-one healthy common carp (weight 78.57 ± 5.99 g and body length 14.24 ± 0.66 cm) were purchased from the Heilongjiang River Fisheries Research Institute, Chinese Academy of Fishery Sciences (Harbin, China).

### 2.2. Animal Rearing and Experimental Design

#### 2.2.1. Animal Rearing

The carp were randomly assigned to 9 tanks (9 per tank), each containing 90 L of dechlorinated tap water. Following a 15-day acclimation period, a 4-OP exposure experiment was conducted. The fish were reared under the following conditions: water temperature at 24 ± 1 °C, pH of 7.2 ± 0.2, dissolved oxygen concentration of 7.2 ± 0.4 mg/L, and a photoperiod of 12 h light/12 h dark. Oxygen was delivered by an oxygen pump. One-third of the water and sponge filters were replaced daily. The carp were fed a commercial diet (Shuyang Wanbing Pet Supplies Co., Ltd., Xingtai, China) twice daily at 8:00 and 17:00.

#### 2.2.2. Experimental Design

The 9 tanks (9 fish per tank) were randomly assigned to 3 groups (3 tanks per group): the control group (untreated dechlorinated tap water), the solvent control group (anhydrous ethanol-supplemented dechlorinated tap water, 33.3 μL/L anhydrous ethanol), and the 4-OP group (4-OP stock solution-supplemented dechlorinated tap water,17 μg/L 4-OP and 33.3 μL/L anhydrous ethanol). The 4-OP concentration (17 μg/L) was set based on the maximum environmental concentration reported in the freshwater environment by Wacheski et al. (2021) [3]. A 4-OP stock solution (510 mg/L) was prepared by dissolving 15.3 mg of 4-OP in 30 mL of anhydrous ethanol. The treatments were as follows: 3 mL of anhydrous ethanol was added to 90 L of water in each tank of the solvent control group, while 3 mL of the 4-OP stock solution was added to 90 L of water in each tank of the 4-OP group. Following the 45-day 4-OP exposure experiment, the carps were euthanized with anesthetic tricaine methane-sulfonate (MS-222, HY-W011777, MedChemExpress, Monmouth Junction, NJ, USA). The anesthetic solution was prepared by dissolving MS-222 in distilled water and buffering to pH 7.0 with sodium bicarbonate. After the fish were euthanized, the heart tissues were taken out immediately.

The heart tissues obtained in the solvent control group were fixed in 4% paraformaldehyde for microstructural observation. The heart tissues obtained in the control and 4-OP groups were divided into four parts for subsequent experiments: the first part was fixed in 4% paraformaldehyde for microstructural observation; the second part was fixed in 2.5% glutaraldehyde solution and was stored at 4 °C for transmission electron microscopy (TEM); the third part was used for tissue homogenate for assay kits; the last part was immediately frozen in liquid nitrogen and stored at −80 °C for quantitative real-time PCR (qRT-PCR) and western blot.

### 2.3. Observation of the Heart Microstructure with Hematoxylin and Eosin Staining

Heart tissues fixed in 4% paraformaldehyde were taken out and rinsed under running water to remove residual fixative [31]. The samples were dehydrated with gradient ethanol (30%, 50%, 70%, 80%, 90%, 95%, and 100%), embedded in paraffin, and sliced into 2–4 μm using microtome (Jinhua Wireless Power Plant, Jinhua, China). The slices obtained were deparaffinized and stained with hematoxylin and eosin (H&E), followed by dehydration and mounting. Finally, the stained slices were scanned using a digital slice scanning system (Shandong Winmedic Technology Co., Ltd., Jinan, China), and microstructural images were visualized and captured using the software ZYFViewer (version 1.0; Shandong Winmedic Technology Co., Ltd., Jinan, China).

### 2.4. Observation of the Heart Ultrastructure with Transmission Electron Microscopy

Heart tissues fixed in 2.5% glutaraldehyde were taken out and rinsed three times (15 min each) with 0.1 M phosphate buffer (pH 7.2). The tissues were fixed again with 1% osmium acid at room temperature for 2 h, followed by three time rinses (15 min each) with 0.1 M phosphate buffer (pH 7.2). The tissues were dehydrated in a graded ethanol series (50%, 70%, 90%, and 100%, 20 min each) and were incubated twice in acetone (10 min each). Subsequently, the samples were gradually permeated with a gradient mixture of pure acetone and embedded solution (1:1 for 40 min; 1:2 for 1.2 h; and 1:3 for 12 h). The tissues were embedded and sliced into 60–80 nm slices using an ultra-thin slicer (LR604, RMC company, San Diego, CA, USA). The slices were double-stained with uranyl acetate and lead citrate. Finally, the heart ultrastructure was observed using a transmission electron microscope (gem-1200es, Tokyo Electronic and Optical Laboratory, Tokyo, Japan).

### 2.5. Determination of Oxidative Stress Markers and ATPase Activities with Assay Kits

Heart tissue (0.1 g) was mixed with physiological saline (0.9 mL) in 1.5 mL tubes and homogenized at 4 °C using a frozen grinder (Hangzhou Suizhen Biotechnology Co., Ltd., Hangzhou, China) to prepare a 10% tissue homogenate. The grinding program was set as follows: 1 min of grinding at 6.5 m/s; 1 min of standing; followed by another 1 min of grinding at 6.5 m/s. The homogenate was then centrifuged at 620 RCF at 4 °C for 10 min. using a refrigerated centrifuge (Mikro 200R, Hettich, Kirchlengern, Germany). The supernatant was collected for the determination of four ATPase activities with ATPase assay kit (A016-2, Na^+^K^+^-ATPase, Ca^2+^Mg^2+^-ATPase, Ca^2+^-ATPase, and Mg^2+^-ATPase), of H_2_O_2_ content with H_2_O_2_ assay kit (A064-1), of CAT activity with CAT assay kit (A007-1), of SOD activity with SOD assay kit (A001-1), and of T-AOC content with T-AOC assay kit (A001-1). The five assay kits were purchased from Nanjing Jiancheng Biotechnology Research Institute (Nanjing, China). All assays were performed in triplicate (*n* = 3).

For the ATPase assay, ATPase catalyzes the hydrolysis of ATP to produce inorganic phosphate, and absorbance was measured at 660 nm using a microplate reader (Molecular Devices Co., Ltd., Shanghai, China). For the H_2_O_2_ assay, H_2_O_2_ reacts with molybdic acid to form a compound, and absorbance was measured at 405 nm. For the CAT assay, CAT decomposes H_2_O_2_, and the reaction is halted by ammonium molybdate being added to form a yellowish compound, and the absorbance was measured at 405 nm. For the SOD assay, SOD inhibits superoxide anions, leading to the formation of nitrite, and the absorbance was measured at 550 nm. For the T-AOC assay kit, the antioxidant reduces Fe^3+^ to Fe^2+^, Fe^2+^ and phenanthroline derivatives form a stable compound, and the absorbance was measured at 520 nm.

### 2.6. Quantitative Real-Time PCR (qRT-PCR) Analysis of Gene Expression

All primers in our experiment were synthesized by Sangon Bioengineering Co., Ltd. (Shanghai, China). The genes detected included 2 internal reference genes U6 (for miR-144) and β-Actin (for mRNA), along with 12 autophagy factors (PI3K, AKT, mTOR, SQSTM1, Beclin1, LC3-I, LC3-II, ULK1, ATG5, ATG12, ATG13, and RB1CC1), 6 mitochondrial dynamics factors (Mff, Drp1, Fis1, Mfn1, Mfn2, and Opa1), AMPK, 5 glycolysis factors (HK1, HK2, LDHA, PGK1, and PGAM2), and 2 oxidative stress factors (Nrf2 and HO-1). The primer sequence information is shown in Table 1.

Heart tissues stored at −80 °C were used for total RNA extraction. The steps for extracting total RNA were as follows: 0.1 g of the tissue was placed into 1.5 mL EP tubes with 1 mL of Trizol reagent (Takara, Kyoto, Japan) and was fully ground at 4 °C using a frozen grinder. The grinding program was set as follows: 1 min of grinding at 6.5 m/s; 1 min of standing; followed by another 1 min of grinding at 6.5 m/s. 400 μL of supernatant was collected and 400 μL of chloroform was added, and the mixture was shaken vigorously and kept at room temperature for 10 min. The mixture was then centrifuged at 14,480 RCF at 4 °C for 15 min using a refrigerated centrifuge (Mikro 200R, Hettich, Kirchlengern, Germany). 400 μL of supernatant was collected and 400 μL of isopropyl alcohol was added. The mixture was inverted gently and kept at room temperature for 10 min, followed by centrifugation at 14,480 RCF at 4 °C for 15 min. The supernatant was discarded, and 1 mL of cold 75% DEPC ethanol was added to precipitate. The mixture was shaken gently and was centrifuged at 10,060 RCF at 4 °C for 10 min. The supernatants were discarded, and the precipitate (the total RNA) was collected. RNA purity was assessed using microspectrophotometer (Hangzhou Allsheng Instrument Co., Ltd., Hangzhou, China). Our obtained ratio of OD260/OD280 was between 1.8 and 2.1, meaning that our RNA purity was reliable.

Total RNA was reverse-transcribed into cDNA with miRcute Plus miRNA First-Strand cDNA kit (B532451, Tiangen Biotechnology Co., Ltd., Beijing, China) and mRNA reverse transcription kit (BL696A, Biosharp, Beijing, China). For miRNA, a 20 μL reaction mixture was prepared containing 10 μL of 2 × miRNA RT Reaction Buffer, 2 μL of miRNA RT Enzyme Mix, and 8 μL of dissolved RNA. For mRNA, a 20 μL reaction mixture was prepared containing 4 μL of 5 × Reaction Mix, 3 μL of Supreme Enzyme Mix, and 13 μL of dissolved RNA. Reverse transcription was performed using a gradient PCR apparatus (Hangzhou, China). The reverse transcription reaction program for miRNA was set as follows: 42 °C for 60 min and 95 °C for 3 min. The reverse transcription reaction program for mRNA was set as follows: 25 °C for 10 min, 55 °C for 15 min, and 85 °C for 5 min. Subsequently, qRT-PCR reaction mixtures were prepared. For miRNA, a 10 μL qRT-PCR reaction mixture was prepared containing 5 μL of 2 × miRcute microRNA Premix (Tiangen Biotechnology Co., Ltd., Beijing, China), 0.2 μL of Forward Primer, 0.2 μL of Reverse Primer, 1 μL of cDNA, and 3.6 μL of RNase-free ddwater. For mRNA, a 10 μL qRT-PCR reaction mixture was prepared containing 5 μL of FastStart Universal SYBR Green Master Kit (Roche Diagnostic GmbH, Mannheim, Germany), 0.3 μL of forward primer, 0.3 μL of reverse primer, 1 μL of cDNA, and 3.4 μL of RNase-free water [32]. The reactions were performed using QuantStudio^®^3 real-time PCR instrument (Roche, Basel, Switzerland). Relative mRNA expression was calculated using the 2 ^−ΔΔCT^ method [15,31]. The experiments were performed in triplicate (*n* = 3).

### 2.7. Western Blot Analysis of Protein Expression

A 1 mL lysis was prepared by mixing 990 μL of western and IP lysis buffer (Beyotime Biotechnology, Nantong, China) with 10 μL of protease inhibitor phenylmethylsulfonyl fluoride (PMSF; Beyotime Biotechnology Nantong, China). Heart tissues stored at −80 °C were used for total protein extraction. The steps for extracting total protein were as follows: 0.1 g of tissue was paced into 1.5 mL EP tubes with 1 mL prepared lysis and was fully ground at 4 °C using a frozen grinder. The grinding program was set as follows: 1 min of grinding at 6.5 m/s; 1 min of standing; followed by another 1 min of grinding at 6.5 m/s. The lysate obtained was centrifuged at 14,480 RCF at 4 °C for 10 min. Protein concentration in the supernatant was determined with a BCA protein concentration assay kit (Beyotime Biotechnology, Nantong, China) in order to calculate the protein loading volume for SDS-PAGE. 600 μL of the supernatant was transferred in new 1.5 mL EP tubes and mixed with 150 μL of SDS-PAGE sample loading buffer (Beyotime Biotechnology, Nantong, China). The mixture was boiled at 98 °C for 10 min to denature proteins and the total protein was obtained.

The preparation involved 4% stacking gels and 12% resolving gels. The protein loading volume was calculated based on the measured protein concentration to perform gel electrophoresis. The conditions for electrophoresis were 80 V for 30 min, followed by 120 V for 1 h and 40 min. After effective separation by molecular weight, proteins on the gels were electroblotted onto PVDF membranes at a constant current (200 mA) using Tris-glycine buffer supplemented with 20% methanol. The membrane was then blocked in 5% non-fat dry milk at 37 °C for 2 h and was subsequently incubated with three primary antibodies (anti-β-actin, anti-SQSTM1, and anti-Beclin1) at 4 °C overnight. Anti-β-actin (WL01372, the dilution ratio is 1:500) and anti-Beclin1 (WL02508, the dilution ratio is 1:500) were from Wanleibio (Shenyang, China). Anti-SQSTM1 (GTX100685, the dilution ratio is 1:500) was bought from GeneTex (San Antonio, TX, USA). The membranes were washed three times (15 min each) with TBST (Servicebio, Wuhan, China), followed by incubation with HRP-conjugated goat anti-rabbit IgG secondary antibody (sc-2004, the dilution ratio is 1:5000; Santa Cruz, CA, USA) at room temperature for 1 h. The membranes were washed three times (15 min each) in TBST. BeyoECL Star (Beyotime Biotechnology, Nantong, China) was dropped on the membranes [33], and protein bands were visualized and captured using Azure c300 chemiluminescence imaging system (Azure Biosystems, Dublin, CA, USA). The gel images were placed as Supplemental Material, as shown in Figure S1. The relative band intensities were quantified using ImageJ software (Version 1.53t) and β-actin was used as the internal reference protein. The experiments were performed in triplicate (*n* = 3).

### 2.8. Statistical Analysis

The obtained data from assay kits, qRT-PCR, and western blot in the control and 4-OP groups were statistically analyzed. IBM SPSS Statistics (version 25, Chicago, IL, USA) was used to analyze the obtained data. The Shapiro–Wilk test was used to assess the normality of the data. Levene’s test was used to verify homogeneity of variance. Subsequently, an independent samples *t*-test was used to analyze the significant differences. Figures were plotted using GraphPad Prism (version 10.0, GraphPad Software, Inc., La Jolla, CA, USA). Data were presented as mean ± standard deviation (SD). * Represented a significant difference (*p* < 0.05). ** Represented an extremely significant difference (*p* < 0.01)

## 3. Results

### 3.1. The Effect of 4-OP Exposure on Heart Tissue Microstructure

**Figure 1:**
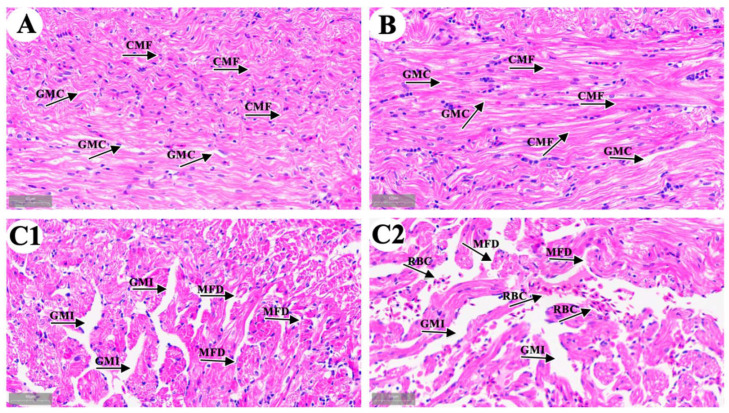
Representative images of common carp heart microstructure. (**A**) control group; (**B**) solvent control group; (**C1**,**C2**) 4-OP group. (**A**,**B**,**C1**,**C2**) (×40). CMF: cardiac muscle fibers; GMC: gaps between myocardial fibers; MFD: myocardial fibers disrupted and disarranged; GMI: gaps between myocardial fibers increased; RBC: red blood cells.

We observed cardiac microstructure in the control group (Figure 1A), the solvent control group (Figure 1B), and the 4-OP group (Figure 1(C1,C2)). In both the control and solvent groups, longitudinal sections of cardiac muscle fibers (CMF) were striped with an orderly arrangement, and the gaps between myocardial fibers (GMC) were uniform. The observation results in the solvent control group indicated that 33.3 μL/L anhydrous ethanol had no harmful effect on common carp hearts. In the 4-OP group, the cardiac muscle fibers were disrupted and disarranged (MFD), and gaps between myocardial fibers were increased (GMI). In addition, as shown in Figure 1(C2), the accumulation of red blood cell (RBC) was observed between myocardial fibers. The observation results in the 4-OP group indicated that exposure to 17 μg/L 4-OP induced cardiac damage in common carp.

### 3.2. The Effect of 4-OP Exposure on Heart Tissue Ultrastructure

**Figure 2:**
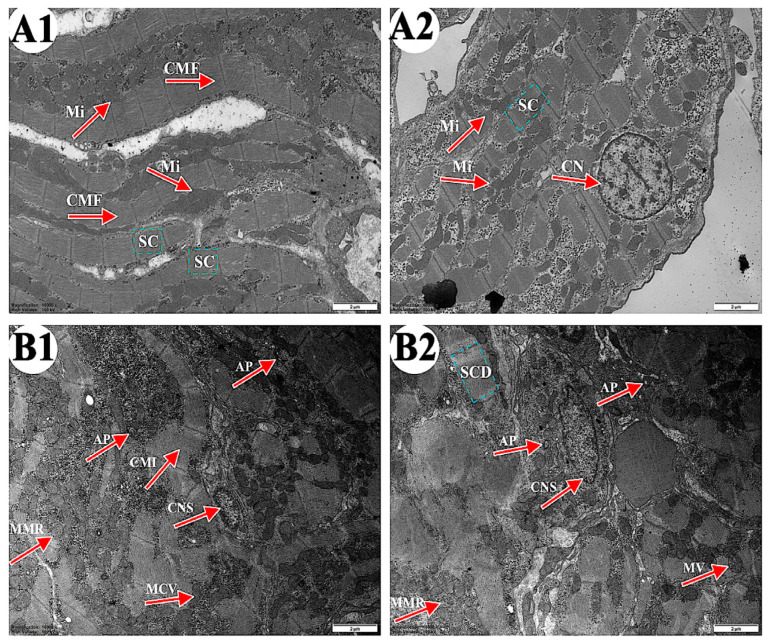
Representative images of common carp heart ultrastructure. (**A1**,**A2**) control groups (×10,000). (**B1**,**B2**) 4-OP groups (×10,000). Magnification: ×15,000. CMF: cardiac muscle fiber; CS: cardiac sarcomere; Mi: mitochondria; CN: cardiomyocyte nucleus; CMI: cardiac muscle fiber irregular; AP: autophagosome; CSD: cardiac sarcomere disorganized; MMR: mitochondrial membrane rupture; MCV: mitochondrial cristae vague; MV: mitochondrial vacuole; CNS: cell nuclei shrink.

As shown in Figure 2, the ultrastructure of carp heart in the control and the 4-OP groups were observed. In the control group (Figure 2(A1,A2)), cardiac muscle fibers (CMF) were arranged in an orderly manner and cardiac sarcomere (CS) displayed a well-organized arrangement. Mitochondria (Mi) were arranged along cardiac muscle fibers and the nucleus of cardiomyocyte nucleus (CN) were rounded. In the 4-OP group (Figure 2(B1,B2)), cardiac muscle fiber appeared irregular (CMI) in arrangement, and cardiac sarcomere was disorganized (CSD). Moreover, autophagosomes (AP), mitochondrial membrane rupture (MMR), mitochondrial cristae vague (MCV), mitochondrial vacuole (MV), and cell nuclei shrink (CNS) were observed.

### 3.3. The Effect of 4-OP Exposure on Autophagy Factors

**Figure 3:**
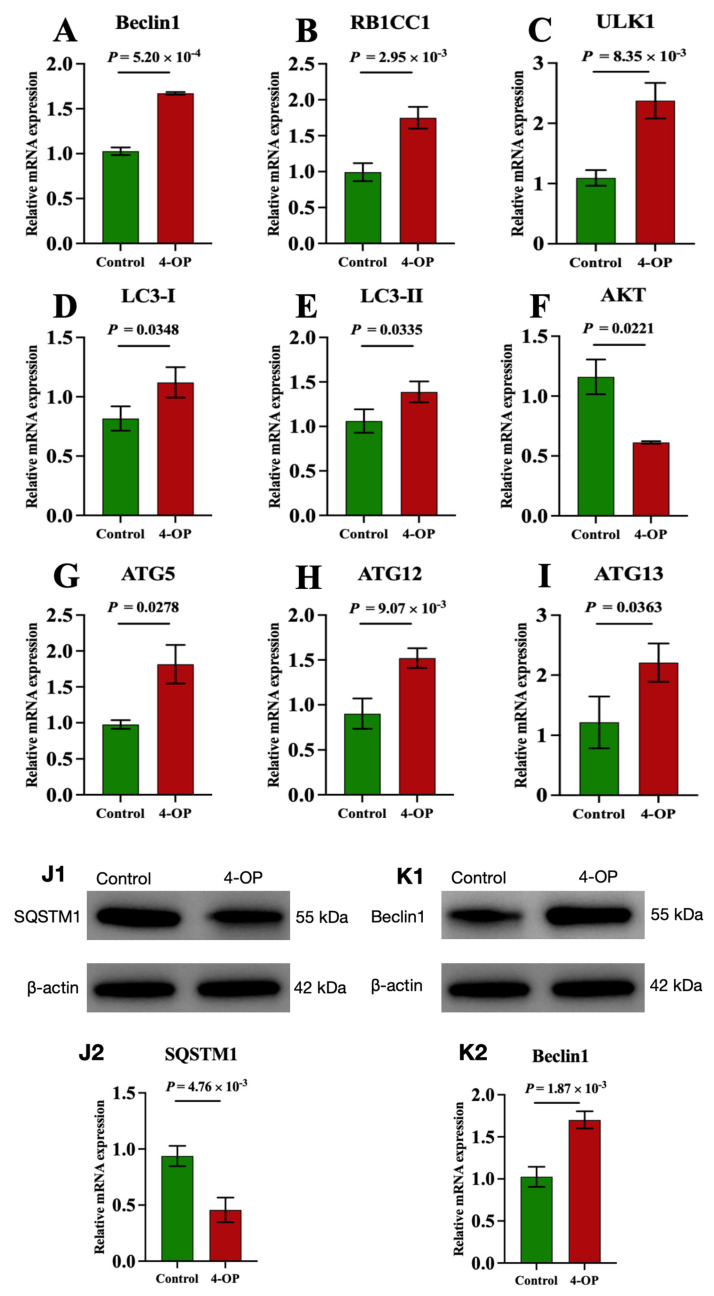
mRNA and protein expressions of autophagy factors in common carp hearts. (**A**) mRNA expression of Beclin1; (**B**) mRNA expression of RB1CC1; (**C**) mRNA expression of ULK1; (**D**) mRNA expression of LC3−I; (**E**) mRNA expression of LC3−II; (**F**) mRNA expression of AKT; (**G**) mRNA expression of ATG5; (**H**) mRNA expression of ATG12; (**I**) mRNA expression of ATG13. (**J1**,**K1**) Representative images of the protein SQSTM1 and Beclin1, respectively; (**J2**,**K2**) Quantization results of protein expression of SQSTM1 and Beclin1, respectively. Each value is represented by mean ± SD. The number of experimental replications was three (*n* = 3).

We investigated the effect of 4-OP on autophagy by measuring mRNA levels of nine autophagy genes (AKT, Beclin1, LC3-I, LC3-II, ULK1, ATG5, ATG12, ATG13, and RB1CC1) and two protein levels of autophagy factors (SQSTM1 and Beclin1), as shown in Figure 3A–K. At the mRNA level, our results revealed that compared with the control group, the mRNA levels of Beclin1 (Figure 3A), RB1CC1 (Figure 3B), ULK1 (Figure 3C), and ATG12 (Figure 3H) in the 4-OP group were extremely significantly (*p* < 0.01) increased, mRNA levels of LC3-I (Figure 3D), LC3-II (Figure 3E), ATG5 (Figure 3G), and ATG13 (Figure 3I) were significantly (*p* < 0.05) increased, while the mRNA level of AKT (Figure 3F) was significantly (*p* < 0.05) decreased. At the protein level, we found an extremely significant (*p* < 0.01) decrease in SQSTM1 (Figure 3(J1,J2)) and an extremely significant (*p* < 0.01) increase in Beclin1 (Figure 3(K1,K2)) following 4-OP treatment.

### 3.4. The Effect of 4-OP Exposure on miR-144 and Its Three Target Genes

**Figure 4:**
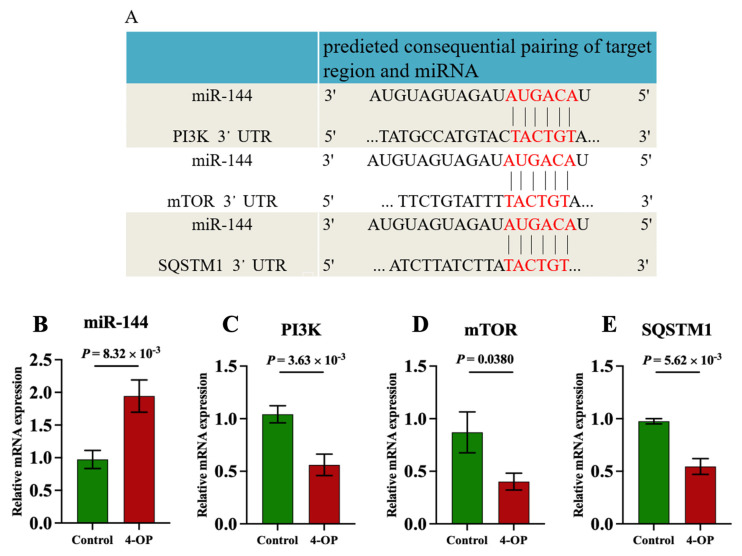
Effects of 4−OP exposure on miR-144 and its three target gene expressions. (**A**) The potential binding site of miR−144 on 3′UTR of PI3K, mTOR, and SQSTM1. (**B**) mRNA level of miR−144. (**C**) mRNA level of PI3K. (**D**) mRNA level of mTOR. (**E**) mRNA level of SQSTM1. Each value is represented by mean ± SD. The number of experimental replications was three (*n* = 3).

Our previous transcriptome sequencing analysis revealed that miR-144 was differentially upregulated, and PI3K, mTOR, and SQSTM1 were differentially downregulated genes following 4-OP exposure. Furthermore, a bioinformatics website (http://www.bioinformatics.com.cn/, accessed on 10 January 2025) was used to predict the potential target genes of miR-144. As shown in Figure 4A, the same six binding sites were identified between miR-144 and the 3′ untranslated regions (3′UTR) of PI3K, mTOR, and SQSTM1, indicating that PI3K, mTOR, and SQSTM1 were target genes of miR-144. Moreover, we further validated that PI3K, mTOR, and SQSTM1 were target genes of miR-144 at transcription levels (Figure 4B–E). The results showed that compared with the control group, the mRNA level of miR-144 (Figure 4B) was extremely significantly (*p* < 0.01) upregulated in the 4-OP group. Compared with the control group, the mRNA level of mTOR (Figure 4D) in the 4-OP group was significantly (*p* < 0.05) downregulated, and the mRNA levels of PI3K (Figure 4C) and SQSTM1 (Figure 4E) in the 4-OP group were extremely significantly (*p* < 0.01) downregulated.

### 3.5. The Effect of 4-OP Exposure on Mitochondrial Fission and Mitochondrial Fusion Factors

**Figure 5:**
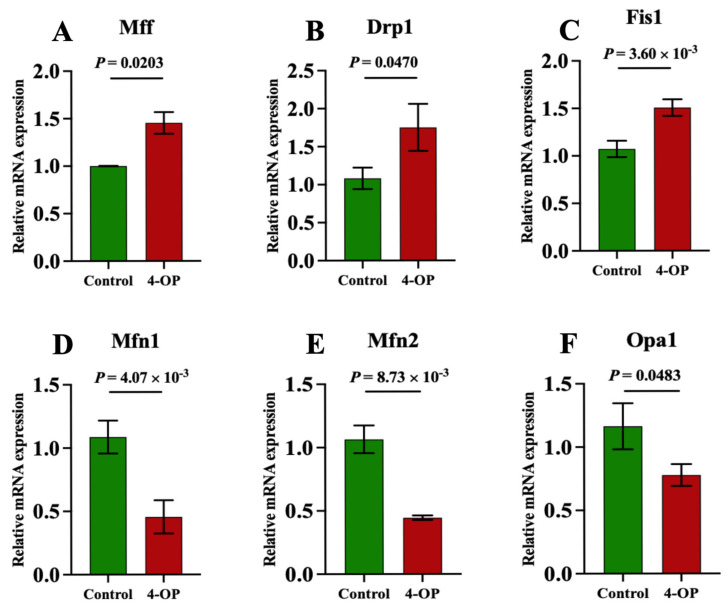
mRNA expression of mitochondrial fission and mitochondrial fusion genes in common carp. (**A**) mRNA expression of Mff; (**B**) mRNA expression of Drp1; (**C**) mRNA expression of Fis1; (**D**) mRNA expression of Mfn1; (**E**) mRNA expression of Mfn2; (**F**) mRNA expression of Opa1. Each value is represented by mean ± SD. The number of experimental replications was three (*n* = 3).

We performed qRT-PCR to assess the expression of genes involved in mitochondrial fission and fusion (Figure 5A–F). Compared with the control group, the mRNA expression of Mff (Figure 5A) and Drp1 (Figure 5B) was significantly (*p* < 0.05) elevated, and the mRNA expressions of Fis1 (Figure 5C) were extremely significantly (*p* < 0.01) elevated following 4-OP treatment. The mRNA expressions of Mfn1 (Figure 5D) and Mfn2 (Figure 5E) were extremely significantly (*p* < 0.01) reduced, and Opa1 (Figure 5F) was significantly (*p* < 0.05) reduced in the 4-OP group compared with the control group.

### 3.6. The Effect of 4-OP Exposure on Energy Metabolism Factors

**Figure 6:**
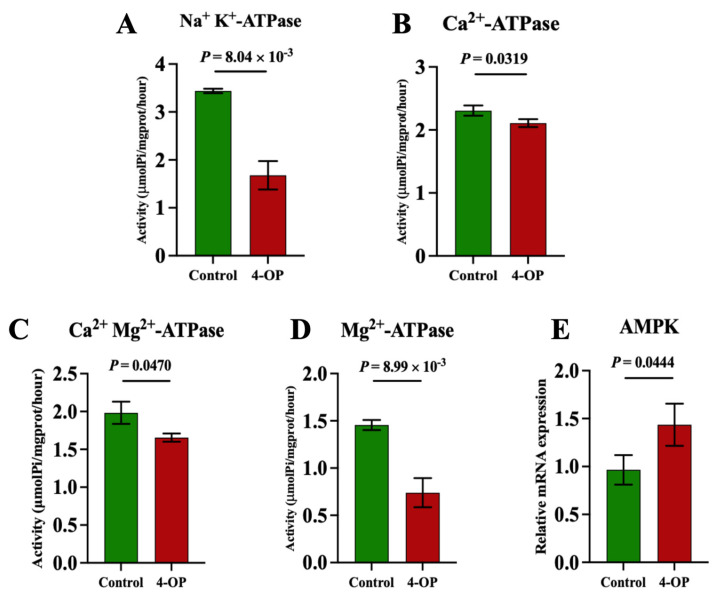
Activities of ATPase and mRNA expression of AMPK in common carp hearts. (**A**) Activity of Na^+^K^+^−ATPase; (**B**) activity of Ca^2+^Mg^2+^−ATPase; (**C**) activity of Ca^2+^−ATPase; (**D**) activity of Mg^2+^−ATPase. (**E**) mRNA expression of AMPK. Each value is represented by mean ± SD. The number of experimental replications was three (*n* = 3).

**Figure 7:**
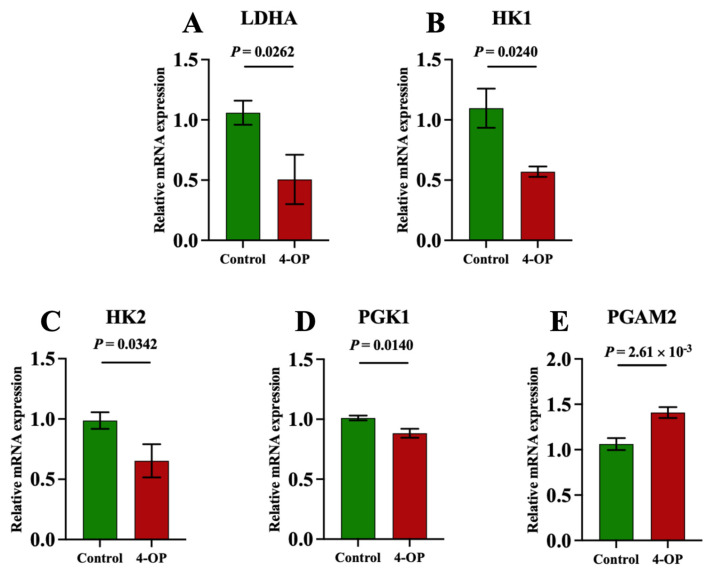
mRNA expression of glycolysis factors in common carps. (**A**) mRNA expression of LDHA; (**B**) mRNA expression of HK1; (**C**) mRNA expression of HK2; (**D**) mRNA expression of PGK1; (**E**) PGAM2 mRNA expression. Each value is represented by mean ± SD. The number of experimental replications was three (*n* = 3).

To investigate whether 4-OP exposure can affect energy metabolism in the hearts of common carp, we assessed four ATPase (Figure 6A–D), AMPK (Figure 6E), and five glycolysis genes (Figure 7A–E). Among the four ATPases, the activities of Na^+^K^+^-ATPase and Mg^2+^-ATPase in the 4-OP group were extremely significant (*p* < 0.01) and lower than those in the control group, and the activities of Ca^2+^Mg^2+^-ATPase and Ca^2+^-ATPase in the 4-OP group were significantly (*p* < 0.05) lower than those in the control group. The mRNA expression of AMPK in the 4-OP group was significantly (*p* < 0.05) higher than that in the control group. For 5 glycolysis genes, the mRNA expressions of LDHA (Figure 7A), HK1 (Figure 7B), HK2 (Figure 7C), and PGK1 (Figure 7D) were significantly (*p* < 0.05) lower than those in the control group, the while mRNA level of PGAM2 (Figure 7E) was extremely significantly (*p* < 0.01) higher than that in the control group.

### 3.7. The Effect of 4-OP Exposure on Oxidative Stress Indexes

**Figure 8:**
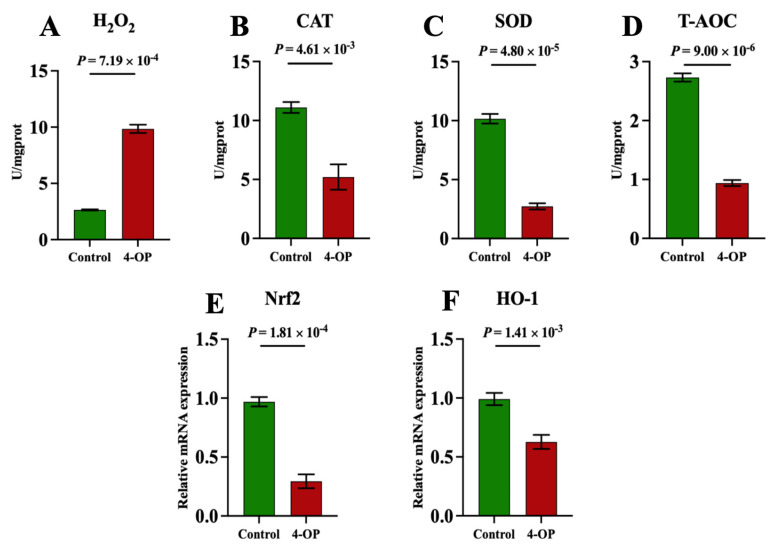
Oxidative stress indexes in common carp hearts. (**A**) H2O2. (**B**) CAT. (**C**) SOD. (**D**) T−AOC. (**E**) mRNA expression of Nrf2. (**F**) mRNA expression of HO−1. Each value is represented by mean ± SD. The number of experimental replications was three (*n* = 3).

To investigate whether 4-OP can induce oxidative stress in common carp hearts, we measured the content of oxidant H_2_O_2_, and the activities of antioxidants CAT, SOD, and T-AOC, as well as the mRNA levels of the antioxidant factors Nrf2 and HO-1, as shown in Figure 8A–F. The H_2_O_2_ (Figure 8A) content was found to be extremely significantly (*p* < 0.01) upregulated in the 4-OP group compared to the control group. CAT (Figure 8B), SOD (Figure 8C), and T-AOC (Figure 8D) activities, as well as the mRNA levels of Nrf2 (Figure 8E) and HO-1 (Figure 8F), were found to be extremely significantly (*p* < 0.01) downregulated in the 4-OP group compared to the control group.

## 4. Discussion

### 4.1. 4-OP Treatment Induced Heart Damage in Common Carp

Two studies reported that 4-OP caused damage in fish. 4-OP exposure impaired primary hepatic cells derived from pearl danios [8], and caused gill damage in common carp [9]. However, no studies have reported whether 4-OP can damage the heart in fish. Microstructure and ultrastructure observation are effective methods to study animal damage caused by environmental stress [15,28,34]. Thus, we observed the microstructure and ultrastructure of carp hearts after 4-OP exposure to investigate whether 4-OP exposure can damage carp hearts. Both our microstructural observation and ultrastructural observation revealed that 4-OP exposure damaged carp hearts. After 4-OP exposure, microstructural observation showed disrupted and disarranged cardiac muscle fibers and increased gaps between myocardial fibers. Ultrastructural observation showed ruptured mitochondrial membrane, vague mitochondrial cristae, shrunken cell nuclei, and autophagosomes. The observation of autophagosomes further revealed that 4-OP exposure induced autophagy and mitochondrial damage in carp hearts. Our findings are supported by two studies showing that harmful endocrine disruptors damaged heart tissue in common carp. A study by Wu et al. (2022) found that exposure to polystyrene nanoplastics increased the gap between cardiomyocytes and broke muscle fibers in common carp hearts [35]. Liu et al. (2023) observed ruptured cardiac muscle fibers, disordered arrangement of cardiac muscle fibers, ruptured mitochondrial cristae, and autophagosomes, and found autophagic damage in common carp hearts following cd treatment [14].

### 4.2. 4-OP Exposure Induced Autophagy

Recent studies have found that autophagy plays a crucial role in the mechanism underlying fish damage caused by toxic environmental pollutants [20,26,28]. PI3K, AKT, mTOR, SQSTM1, Beclin1, LC3-I, LC3-II, ULK1, ATG5, ATG12, ATG13, and RB1CC1 are autophagy factors. PI3K, AKT, and mTOR are negative regulators of autophagy. Beclin1, ATG5, ATG12, LC3I, and LC3II contribute to autophagosome assembly, with LC3-II recruited to the autophagosomal membrane to facilitate autophagosome formation and initiate autophagy. ULK1 participates in autophagy initiation by forming a complex with ATG13 and RB1CC1 [36]. SQSTM1 (also known as p62), an autophagy substrate protein, acts as a negative regulator of autophagy. In our study, we found that exposure to 4-OP increased the levels of ULK1, Beclin1, ATG5, ATG12, ATG13, RB1CC1, LC3-I, and LC3-II and decreased the levels of PI3K, AKT, mTOR, and SQSTM1, which indicated that 4-OP treatment induced autophagy in common carp hearts. Other experiments are consistent with our findings. Liu et al. (2022) reported that cobalt chloride upregulated the levels of ATG5, RB1CC1, LC3I, and LC3-II, and induced autophagy in mouse kidneys [37]. In MEHP-treated CIK cells, Beclin1, ULK1, LC3-II, Atg12, and Atg13 increased, and autophagy occurred [20]. Exposure to synthetic phenolic antioxidants upregulated the levels of Beclin1, LC3-I, and ATG13, and downregulated the levels of PI3K, AKT, and mTOR, and induced autophagy in grass carp livers [28]. After 2,4-di-tert-butylphenol exposure, mTOR and SQSTM1 decreased, Beclin1, LC3-I, LC3-II, and ATG5 increased, and autophagy occurred in common carp livers [26]. Moreover, it was discovered that LY294002 (PI3K inhibitor) treatment inhibited the level of PI3K, as well as the levels of AKT and mTOR in human breast cancer cells [38]. Zhao et al. (2021) demonstrated that MK2206 (AKT inhibitor) treatment inhibited the levels of Akt and mTOR and aggravated stigmasterol-induced autophagy in gastric cancer cells [39]. mTOR agonist MHY1485 alleviated hypoxia-induced mTOR reduction, ULK1 elevation, and autophagy in the pulmonary artery cells of rats [40]. Therefore, our results indicated that the PI3K-AKT-mTOR/ULK1 pathway took part in 4-OP-induced autophagic damage in common carp hearts. For the first time, we found that the PI3K-AKT-mTOR/ULK1 pathway was involved in the molecular mechanism of autophagy caused by estrogen, and further research is needed.

### 4.3. miR-144 Mediated 4-OP-Induced Autophagy

miRNAs are key regulators of various biological processes, such as autophagy. Three studies reported that miRNAs mediated autophagy caused by toxic endocrine disruptors. miR-30a was shown to mediate estrogen diethylstilbestrol exposure-caused autophagy via targeting Beclin1 in mouse T cell lymphoma cell line (EL4) [41]. miR-155 mediated autophagy by targeting PI3K in polychlorinated biphenyls (PCBs)-exposed rat chondrocytes [42]. Zhou et al. (2023) found that miR-144 mediated autophagy in rat ovarian cells treated with 4-vinylcyclohexene diepoxide by targeting PTEN [43]. However, whether miRNAs were involved in 4-OP exposure-induced autophagy in common carp hearts was still unknown. miRNAs regulate biological processes mainly through binding the 3′UTR of target mRNAs. Thereby, a bioinformatics website was used in our study and we found six binding sites between miR-144 and the 3′UTR of PI3K, mTOR, and SQSTM1 genes, predicting that PI3K, mTOR, and SQSTM1 are target genes of miR-144. Furthermore, we measured miR-144, PI3K, mTOR, and SQSTM1 at the transcriptional level and found that 4-OP treatment increased the level of miR-144 and decreased the levels of PI3K, mTOR, and SQSTM1 in common carp hearts, further demonstrating that PI3K, mTOR, and SQSTM1 were target genes of miR-144. The findings indicated that miR-144 mediated 4-OP-induced autophagy in common carp hearts via targeting PI3K, mTOR, and SQSTM1. Two studies support the targeted relationship of our study. Ren et al. (2018) confirmed that mTOR was a direct target of miR-144 through a luciferase reporter assay and overexpression experiment; miR-144 overexpression increased the level of miR-144 and reduced the level of mTOR in osteosarcoma cells [44]. Similarly, Wang et al. (2019) reported that miR-144 overexpression upregulated the level of miR-144 and downregulated the level of PI3K in SW1990 cells [45]. Notably, based on our above finding regarding the PI3K-AKT-mTOR/ULK1 pathway, our results indicated that the miR-144/PI3K-AKT-mTOR/ULK1 pathway participated in 4-OP-induced autophagy in common carp hearts. To our knowledge, for the first time, we found that miR-144 targeted three genes PI3K, mTOR, and SQSTM1 to mediate autophagy in common carp hearts following 4-OP exposure and the miRNA-144/PI3K-AKT-mTOR/ULK1 pathway took part in the molecular mechanism underlying toxic environmental pollutant-caused autophagic damage in animals, which needs to be explored in the future.

### 4.4. 4-OP Exposure Caused Energy Metabolism Disorder

The heart maintains blood circulation mainly through energy generated by ATP hydrolysis, a process catalyzed by ATPase that converts ATP into ADP to release energy. Four ATPases—Na^+^K^+^-ATPase, Ca^2+^Mg^2+^-ATPase, Ca^2+^-ATPase, and Mg^2+^-ATPase—play important roles in the process. Three reports found that environmental pollutants caused decreases in the four ATPases and energy metabolism disorder in fish [13,14,46]. In monobutyl phthalate-treated zebrafish, Na^+^-K^+^-ATPase, Ca^2+^-ATPase, and Mg^2+^-ATPase decreased and energy metabolism was disturbed in gills [13]. Cui et al. (2023) found that chlorpyrifos treatment decreased the activities of four ATPases (Na^+^K^+^-ATPase, Mg^2+^-ATPase, Ca^2+^-ATPase, and Ca^2+^Mg^2+^-ATPase) and disrupted energy metabolism in common carp livers [46]. After Cd treatment in common carp, Na^+^K^+^-ATPase, Ca^2+^Mg^2+^-ATPase, Ca^2+^-ATPase, and Mg^2+^-ATPase decreased and energy metabolism impairment occurred in hearts [14]. Thus, we hypothesized that 4-OP can reduce the four ATPases in common carp hearts. We measured the activities of four ATPases and found that 4-OP exposure reduced the activities of the four ATPases, indicating that 4-OP caused energy metabolism disorder in common carp hearts.

Glycolysis is one of the major energy metabolism pathways, generating ATP through the breakdown of glucose. HK1, HK2, PGAM2, LDHA, and PGK1 are glycolysis enzymes. HK1 is a first-rate-limiting enzyme in glycolysis. The knockdown of HK1 induced energy metabolism disorders and accelerated the invasion of cervical cancer HeLa cells [47]. HK2 is a key enzyme in glucose metabolism. HK2 depletion suppressed glycolysis and led to energy impairment in mice glioma cells [48] (Hu et al., 2022). Mikawa et al. (2021) reported that the overexpression of PGAM2 inhibited glycolysis and reduced glucose metabolism in mouse cells [49]. It has been found that knockdown LDHA impaired glucose metabolism in Hep3B cells [50]. PGK1 silencing inhibited glycolysis in mouse glioma cells [51]. Therefore, we detected the five glycolysis indicators and further investigated whether toxic substance 4-OP can induce energy metabolism disorders from the perspective of glycolysis. In our experiment, HK1, HK2, LDHA, and PGK1 mRNA expression decreased, while PGAM2 mRNA expression increased after 4-OP treatment, indicating that 4-OP induced glycolysis disorders in common carp hearts. Similar studies supported our findings. Bisphenol AF exposure decreased the levels of HK1 and HK2, and inhibited glycolysis in zebrafish (*Danio rerio*) muscle [17]. In Cd-treated largemouth bass (*Micropterus salmoides*), LDHA decreased [16]. In addition, two studies have found that endocrine disruptors increased AMPK and induced energy metabolism disorder in fish. In nonylphenol-treated zebrafish, AMPK increased and energy metabolism disorder occurred [15]. Cui et al. (2023) found that chlorpyrifos exposure increased AMPK and induced energy metabolism disorder in common carp livers [46]. However, it was unknown if AMPK can take part in the molecular mechanism of 4-OP treatment-caused energy metabolism disorder in common carp. In our experiment, we found that AMPK increased after 4-OP treatment, confirming that 4-OP disrupted energy metabolism in common carp hearts. Interestingly, it was found that treatment of duck embryonic cells with compound C (an AMPK inhibitor) reduced AMPK, increased mTOR, and inhibited autophagy [52]. AMPK activator O304 activated AMPK, reduced the level of mTOR, and caused autophagy in mouse kidney cells [53] (Zhu et al., 2022). Therefore, our findings also suggested that energy metabolism disorder mediated autophagy via AMPK/mTOR axis in the context of 4-OP poisoning in common carp hearts, which requires further investigation.

### 4.5. 4-OP Treatment Induced Mitochondrial Damage

Mitochondria are essential organelles that serve as the primary energy producers in cells, supplying a large amount of energy to sustain continuous systolic and diastolic functions of the heart. The functional state of mitochondria directly determines cardiac energy supply efficiency. Therefore, we wanted to investigate whether 4-OP exposure can damage mitochondria in common carp hearts. Our morphological observation showed that 4-OP caused cardiac mitochondrial damage in carp, as evidenced by mitochondrial vacuole and mitochondrial crest fracture. Several studies reported that six factors (Mff, Drp1, Fis1, Mfn1, Mfn2, and Opa1) were involved in the mechanism of mitochondrial damage. Mfn1 and Mfn2 knockout in mice cardiomyocytes and Opa1 knockout in mice embryonic fibroblasts resulted in mitochondrial damage [54,55,56]. The overexpression of Mff induced mitochondrial fragmentation in HeLa cells [57]. Treatment with Mdivi-1 (a Drp1 inhibitor) alleviated mitochondrial fragmentation in mouse mammary epithelial cells [58]. Fis1 knockout mitigated palmitic acid-induced mitochondrial damage in rat primary cardiomyocytes [59]. Mitochondrial dynamics are regulated by two opposing processes: fission and fusion. Mff, Drp1, and Fis1 are mitochondrial fission proteins, while Mfn1, Mfn2, and Opa1 are mitochondrial fusion proteins. In our experiment, we measured the six factors and found that exposure to 4-OP increased the levels of three mitochondrial fission factors (Mff, Drp1, and Fis1) and decreased the levels of three mitochondrial fusion factors (Mfn1, Mfn2, and Opa1). The results indicated that 4-OP exposure disrupted the balance between mitochondrial fission and fusion, suggesting that 4-OP induced mitochondrial dynamics imbalance and damage in common carp hearts. Previous studies demonstrated that toxic endocrine disruptors disrupted mitochondrial dynamics through similar mechanisms. Fan et al. (2024) reported that dibutyl phthalate exposure in zebrafish increased the level of Fis1 and decreased the levels of Mfn1, Mfn2, and Opa1, and damaged mitochondria [21]. Liu et al. (2023) found that Cd treatment upregulated Mff and Drp1 while it downregulated the levels of Mfn1, Mfn2, and OPa1, and caused the imbalance between mitochondrial fission and fusion mitochondrial dynamics imbalance and damage in common carp hearts [14]. Notably, Peng et al. (2022) demonstrated that Mff knockdown decreased the level of Drp1 in the 12-SV40 human corneal epithelial cell line [60]. Therefore, our results suggested that the Mff-Drp1 axis was involved in 4-OP-caused mitochondrial dynamics imbalance. Interestingly, Yuan et al. (2023) found that the transfection of siRNA-Mfn2 into SH-SY5Y cells reduced the protein expression of SQSTM1 and increased the protein expression of Beclin1 [61]. The knockdown of Mff depressed the level of LC3-II in the 12-SV40 human corneal epithelial cell line [60]. Therefore, our data suggested that mitochondrial dynamics imbalance mediated autophagy through the Mfn2-SQSTM1, Mfn2/Beclin1, and Mff-LC3-II axes in the hearts of 4-OP exposed common carp. In addition, treatment with AICAR (AMPK activator) increased the levels of AMPK and Mff in bovine luteal cells [62]. Therefore, our results suggested that energy metabolism disruption mediated mitochondrial damage via the AMPK-Mff-Drp1 pathway in common carp hearts. Energy metabolism disorder took part in mitochondrial fission and fusion imbalance-mediated autophagy through the AMPK-Mff-LC3-II pathway. For the first time, we found that the AMPK-Mff-LC3-II pathway partook in the molecular mechanism of animal poisoning, and our new finding needs to be further investigated.

### 4.6. Exposure to 4-OP Caused Oxidative Stress

Two studies found that toxic environmental pollutants induced oxidative stress, autophagy, energy metabolism disorder, and mitochondrial dynamics imbalance in fish. Oxytetracycline exposure induced oxidative stress, mitochondrial dynamics imbalance, and autophagy in largemouth bass (*Micropterus salmoides*) livers [63]. Cd exposure induced oxidative stress, autophagy, energy metabolism disorder, and mitochondrial dynamics imbalance in common carp hearts [14]. However, whether 4-OP can induce oxidative stress in common carp hearts was still unknown. Therefore, we wanted to explore whether 4-OP exposure can induce oxidative stress in common carp hearts. Oxidative stress arises from an imbalance between cellular antioxidants and oxidants. H_2_O_2_ is an oxidant factor, and CAT, SOD, T-AOC, Nrf2, and HO-1 are antioxidant factors. H_2_O_2_ is an aerobic metabolite and induces oxidative stress. CAT, an antioxidant enzyme, degrades H_2_O_2_ into H_2_O and oxygen. Antioxidant agent SOD catalyzes the dismutation of superoxide radicals into hydrogen peroxide (H_2_O_2_) and ordinary molecular oxygen (O_2_) or hydrogen peroxide (H_2_O_2_) for scavenging free radicals in the body. T-AOC reflects the total antioxidant capacity of the body. Nrf2 and HO-1 play a role in activating the antioxidant response to defend against oxidative stress. Therefore, we detected oxidant H_2_O_2_ and five antioxidant factors (CAT, SOD, T-AOC, Nrf2, and HO-1), and found that 4-OP exposure increased H_2_O_2_ while reducing the five antioxidant factors. The results suggested that 4-OP led to oxidative stress by disrupting the balance between antioxidants and oxidants in common carp hearts. Several studies support our results. 4-tBP exposure upregulated H_2_O_2_, downregulated CAT, SOD, and T-AOC, and induced oxidative stress in common carp head kidneys [23]. An increase in H_2_O_2_ and decreases in CAT and SOD were found in Cd-induced oxidative stress in common carp hearts [14]. Desai et al. (2023) demonstrated that Nrf2 decreased and oxidative stress occurred in the brain of zebrafish treated with 4-nonylphenol [24]. In 1,2,3,7,8-polychlorinated dibenzo-p-dioxin-treated primary Atlantic cod hepatic cells, the level of HO-1 decreased and induced oxidative stress [25]. Additionally, Xu et al. (2022) reported that H_2_O_2_ treatment disturbed mitochondrial dynamics and caused mitochondrial damage by increasing Drp1 and decreasing Mfn2 in rat retinal neuronal cell lines [64]. Therefore, our results indicated that oxidative stress mediated mitochondrial dynamics imbalance via the H_2_O_2_-Drp1 and H_2_O_2_/Mfn2 axes following 4-OP exposure, and that oxidative stress triggered 4-OP-induced autophagy mediated by mitochondrial dynamics imbalance. In addition, exposure to H_2_O_2_ upregulated the AMPK in MLO-Y4 cells [65] (Wei et al., 2023). Thereby, our findings indicated that oxidative stress mediated energy metabolism disorder via the H_2_O_2_-AMPK axis in common carp hearts. Therefore, our findings indicated that oxidative stress triggered energy metabolism disorder, induced mitochondrial dynamics imbalance, and caused autophagy via the H_2_O_2_-AMPK-Mff pathway.

## 5. Conclusions

In conclusion, our results demonstrated that the heart was a target organ of 4-OP and that 4-OP exposure led to mitochondrial damage, autophagy, and damage in common carp hearts. 4-OP exposure increased the levels of miR-144, Beclin1, RB1CC1, ULK1, LC3-I, LC3-II, ATG5, ATG12, and ATG13, and decreased the levels of PI3K, AKT, mTOR, and SQSTM1, and induced autophagy. miR-144 mediated autophagy via targeting PI3K, mTOR, and SQSTM1, and the miR-144/PI3K-AKT-mTOR/ULK1 pathway may took part in 4-OP induced autophagy. In addition, 4-OP exposure upregulated mitochondrial fission genes (Mff, Drp1, and Fis1) and downregulated mitochondrial fusion genes (Mfn1, Mfn2, and Opa1), and resulted in an imbalance between mitochondrial fission and mitochondrial fusion and mitochondrial dynamics imbalance, as well as mitochondrial damage. The Mff-Drp1 axis was involved in 4-OP-caused mitochondrial dynamics imbalance. Mitochondrial dynamics imbalance mediated autophagy via the Mfn2-SQSTM1, Mfn2/Beclin1, and Mff-LC3-II axes. Interestingly, 4-OP induced energy metabolism disorder, as evidenced by the decrease in four ATPases (Na^+^K^+^-ATPase, Ca^2+^Mg^2+^-ATPase, Ca^2+^-ATPase, and Mg^2+^-ATPase), the increases in four glycolytic enzymes (HK1, HK2, LDHA, and PGK1), the decrease in glycolytic enzyme PGAM2, and the increase in energy sensor AMPK. Energy metabolism disorder mediated 4-OP-caused mitochondrial dynamics imbalance via the AMPK-Mff-Drp1 pathway, and energy metabolism disorder triggered mitochondrial dynamics imbalance-mediated autophagy induced by 4-OP via the AMPK-Mff-LC3-II pathway. We also found that exposure to 4-OP increased H_2_O_2_, decreased CAT, SOD, T-AOC, Nrf2, and HO-1, and resulted in oxidative stress. Oxidative stress mediated energy metabolism disorder via the H_2_O_2_-AMPK axis. Therefore, our results suggested that oxidative stress triggered energy metabolism disorder, induced mitochondrial dynamics imbalance, and caused autophagy via the H_2_O_2_-AMPK-Mff-LC3-II pathway. Our study provided new insights into the molecular mechanism of 4-OP-induced toxicity in common carp and provided potential targets and new information on endocrine disruptors-induced cardiotoxicity in fish. However, whether 4-OP is toxic to other fish species remains unclear, which is a limitation of our study and requires further investigation in the future.

## Figures and Tables

**Table 1:** Primer sequences for qRT-PCR.

Gene	Accession Number	Forward (5′→3′)	Reverse (5′→3′)
U6	XM_019117289	CCTCGCTTCGGCAGCACATATAC	Provided by Tiangen Biotech Co., Ltd.
miR-144	MI0023330	GCGGCGCCTACAGTATAGATGATGT	Provided by Tiangen Biotech Co., Ltd.
β-actin	M24113	ATGGACTCTGGTGATGGTGTGAC	TTTCTCTTTCGGCTGTGGTGGTG
PI3K	BG936137	AGTCAGTGCCTGTGGCTGAG	CGTGTCCATGACCTCAGAGC
mTOR	XM_042758124	AGCAGTATGGAGGGAGAGCGTATG	AGCAGTATGGAGGGAGAGCGTATG
P62/SQSTM1	XM_042737795	AAGACCAAGGCAGTGATGAGGAATG	GCTTGTGCTGGAGTCGGTACTTAG
AKT	XM_042727873	CCTGGTGATGAAGGAGCTGA	CTGTCAGAGAGCCTCCAGCA
ULK1	XM_042748518	GTCATGTGCCAGTAGTTACGCTCAG	CGACACTTGTAGGTTCTGCTCCATC
ATG13	XM_042727972	CATCCTGTAGCAGCAAGGTGAAGAC	CTGGTTGTTGTCGCCTGAGTGG
RB1CC1/FIP200	XM_009302198	CCAGCAGGTCCAGCAGAAGAATG	TTCATGCCGATCCACAAGTTCAGAG
Beclin1	XM_019078401	AGCGTGGACAATCAGATGCGTTAC	TGTTCCAAACTGCCCACTATGCC
ATG5	XM_019082404	ATGTGCGGAAGATGAGCCAAAGAG	GGTGCTGGGATGATGCTGATGTG
ATG12	XM_042740678	ACAGTACAGTCACTCGCTCA	AAAACACTCGAAAAGCACACC
LC3-I	XM_019109703	TGTCAATCAGCACAGCATGGTCAG	AGGTCTCCTGGGAGGCGTAAAC
LC3-II	NM_199604	CTGGGCTCCACAGTACGAAG	CCGCTGCTCAAATGTCCTCC
HK1	XM_042736852	GTCTCGCAGCGTCTCATC	TTTCCATTTCTGTTTCCCTA
HK2	XM_042753262	CATGCAGAGCGTCAGCGTATCC	AAGCCTCGGTTCATCTCCTCCTC
LDHA	XM_042722014	AAGAACCTACGCAAGTGTCATCCAG	GCAAGGCACGCTGAGGAAGAC
PGK1	XM_042747513	CCAGACCCATCCATCCTG	ATTGGCACTTCCCTATTCG
PGAM2	XM_042747782	ACCACGCAGGCTGTTTCC	CATCCCACCTCCACCCAT
AMPK	XM_019104472	ACCAAGTTATCAGCACACCGACAG	ACGCCTGCTCTCCTTCTCATCC
Mfn1	XM_019102515	AGAGACGGGTAAGAGCGTGA	TCCTCCAGAGAAACCACCCT
Mfn2	XM_019065714	ACGTCACCACCTAGCCAAC	TAGCCATCAAATGTGGGCG
Opa1	XM_019105838	CAGTGGGATGCCGCTATACAGTTC	GGTGTGCTGCTCTGGTGTTCG
Drp1	XM_019076184	CGAACTACGTTGTTGCGCT	GAACCCGACTCTGCGTTCTC
Fis1	XM_019093606	CGTACTCTGCTGAAGAATGAACCAG	ACACCTAATCCGATACCGCCAAC
Mff	XM_019117476	CGGCGTTCTCTCTTTCATCCAGTC	GGCTTGCTGCGGTGGTTCTC
Nrf2	XM_042730726	ACATCCCTCTATGCTCCTGACACC	CGTTGCCTCTACAGCCTCAGATTG
HO-1	XM_042757461	ACCAGAAAGGACAGATCACGCAAAC	TGAGGGAAGTAGATGGGCTGAACC

## Data Availability

The data presented in this study are available on request from the corresponding author. The data are not publicly available due to privacy or ethical restrictions.

## Supplementary Materials

The following supporting information can be downloaded at: https://www.mdpi.com/article/10.3390/metabo15060391/s1, Figure S1: The gel images of three biological replicates for β-actin, SQSTM1, and Beclin1; Table S1: Transcriptome sequencing result of common carp under 4-OP exposure.

## Abbreviations

Abbreviations	Original words
4-OP	4-Octylphenol
HK1	hexokinase 1
HK2	hexokinase 2
LDHA	lactate dehydrogenase A
PGK1	phosphoglycerate kinase 1
PGAM2	phosphoglycerate mutase 2
AMPK	AMP-activated protein kinases
PI3K	phosphatidylinositol 3-kinase
AKT	Akt kinase
mTOR	mechanistic target of rapamycin kinase
ULK1	unc-51 like autophagy activating kinase 1
ATG13	autophagy related 13
RB1CC1/FIP200	RB1 inducible coiled-coil 1
P62/SQSTM1	sequestosome 1
Beclin1	beclin 1
ATG5	autophagy related 5
ATG12	autophagy related 12
LC3-I	Microtubule-associated protein 1A/1B-light chain 3-I
LC3-II	Microtubule-associated protein 1A/1B-light chain 3-II
Mfn1	mitofusin 1
Mfn2	mitofusin 2
Opa1	OPA1 mitochondrial dynamin like GTPase
Drp1	Dynamin-related protein 1
Fis1	fission, mitochondrial 1
Mff	mitochondrial fission factor
