# Supplementary material for: Oxidative Stress, Energy Metabolism Disorder, Mitochondrial Damage, and miR-144 Participated in Molecular Mechanisms of 4-Octylphenol-Caused Cardiac Autophagic Damage in Common Carps (Cyprinus carpio L.)
Source: Metabolites. 2025 Jun 11;15(6):391. doi: 10.3390/metabo15060391 (PMC12195614; doi:10.3390/metabo15060391)
Supplement: Supplementary file 1 [file metabolites-15-00391-s001.zip › Table S1.docx]

Table S1. Transcriptome sequencing result of common carp under 4-OP exposure

| miRNA ID | baseMean | log2FC | pvalue | padj | Direction |
| --- | --- | --- | --- | --- | --- |
| ccr-miR-144 | 2703.060966 | 1.414499099 | 1.51749E-06 | 0.000121131 | Up |
