# Supplementary material for: Oxidative Stress, Energy Metabolism Disorder, Mitochondrial Damage, and miR-144 Participated in Molecular Mechanisms of 4-Octylphenol-Caused Cardiac Autophagic Damage in Common Carps (Cyprinus carpio L.)
Source: Metabolites. 2025 Jun 11;15(6):391. doi: 10.3390/metabo15060391 (PMC12195614; doi:10.3390/metabo15060391)
Supplement: Supplementary file 1 [file metabolites-15-00391-s001.zip › Figure S1.pdf]

## Replicate 1

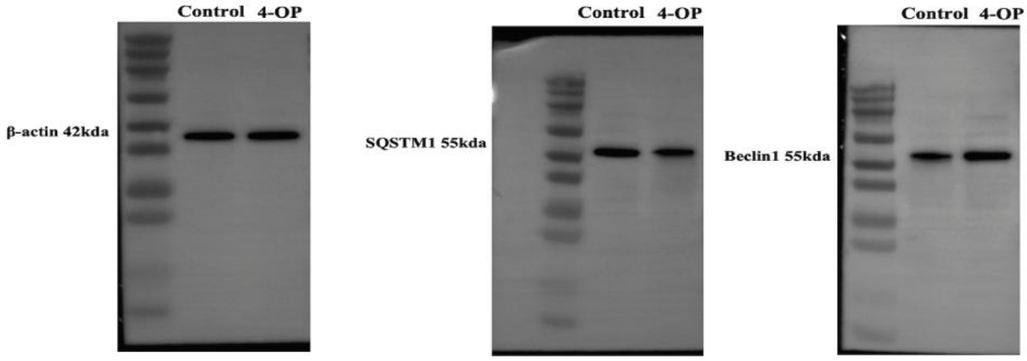


## Replicate 2

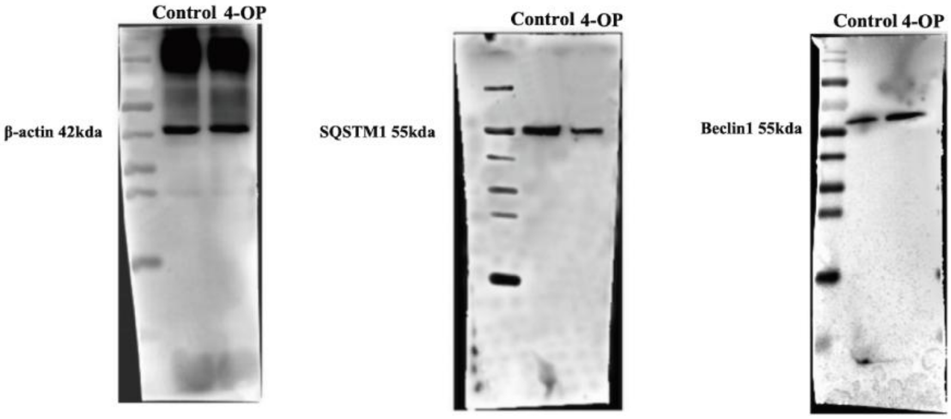


## Replicate 3

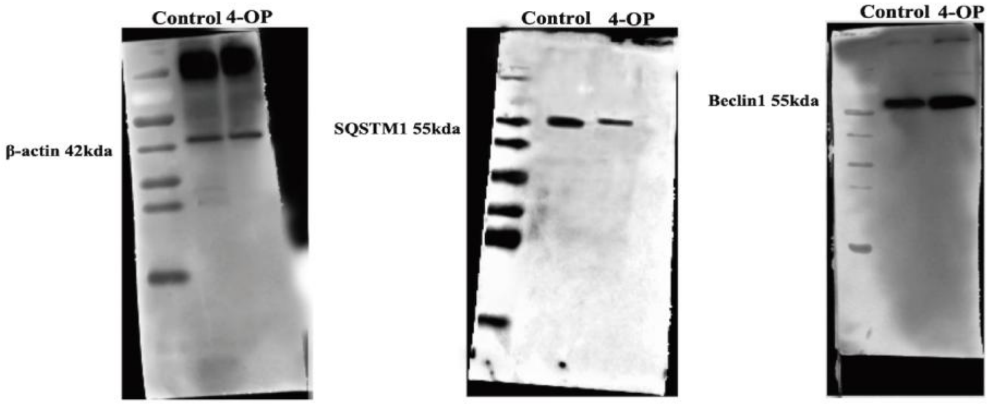


Fig.S1: The gel images of three biological replicates for β-actin, SQSTM1, and Beclin1
